# Supplementary material for: A Novel Peptide with Antifungal Activity from Red Swamp Crayfish Procambarus clarkii
Source: Antibiotics (Basel). 2022 Dec 10;11(12):1792. doi: 10.3390/antibiotics11121792 (PMC9774249; doi:10.3390/antibiotics11121792)
Supplement: Supplementary file 1 [file antibiotics-11-01792-s001.zip › antibiotics-2028371-supplementary.pdf]

Supplementary Material File S1:

LC-MS/MS data were analyzed by the PEAKS software package, that incorporates the de novo sequencing results into the database search.
Database search didn't match any peptide derived from *Crustacea* proteins. However, from the MS/MS spectra, de novo sequencing derived the amino acid sequence of fourteen peptides that remained unidentified by the database search algorithm. In detail, ten peptides were identified in polypeptide-enriched extract from haemocytes and four in polypeptide-enriched extract from haemolymph. Taking into account the characteristics of these peptides, some of them may represent potential AMPs.

nLC-MS/MS of peptide fraction from haemocytes and haemolimph

List of the fourteen de novo derived sequences remained unidentified by the database search algorithm. Peptides #1-#10 were identified in haemocytes extract; peptides #11-#14 were identified in haemolymph extract.

N.	Peptide	ALC (%) [a)]	Experimental m/z	z	Experimental Mass (monoisotopic)	Theoretical Mass (monoisotopic)	Theoretical $m/z=(M+2H^{+})^{2+}$ (monoisotopic)	Error (ppm)[b)]
#1	M(+15.99)FLHGHAV [c)]	90	464.2328	+2	926.4510	926.4433	464.5546	-8.5
#2	E(-18.01)GLDDDERL [d)]	89	522.2357	+2	1042.4568	1042.4567	522.2356	0.1
#3	SSGYGGYGGGRF	87	582.7574	+2	1163.5002	1163.4496	582.7571	0.6
#4	LNVQAQMLLQ	85	579.3215	+2	1156.6284	1156.6274	579.3210	0.9
#5	NNWTGADCKAATLK	84	746.8683	+2	1491.7220	1491.7140	746.8643	5.3
#6	SHGDSALSSTF	82	554.7498	+2	1107.4850	1107.4833	554.7489	1.7
#7	YGGYFGNR	81	467.2144	+2	932.4142	932.4141	467.2143	0.2
#8	ETEASLTAALPRW	81	722.8807	+2	1443.7468	1443.7358	722.8752	7.7
#9	LVDSNGALLDELPVAR	80	841.4600	+2	1680.9054	1680.9047	841.4596	0.5
#10	KLLLDNSAEDLEELASHK	80	1013.0297	+2	2024.0448	2024.0426	1013.0286	1.1
#11	AADSFGETFAATL	92	650.8069	+2	1299.5992	1299.5983	650.8064	0.8
#12	FDTLSSHLVATD	91	653.3208	+2	1304.6270	1304.6249	653.3197	1.8
#13	ETAPLSGVCF	84	512.2412	+2	1022.4678	1022.4743	512.2444	-6.2
#14	FSLVNAVDQTT	80	597.8040	+2	1193.5934	1193.5928	597.8037	0.6

a) PEAKS assigns a local confidence score for each amino acid in a de novo sequence. The local confidence score ranges from 0% to 99%. indicating how confident the algorithm considers a particular amino acid as the correct assignment. Moreover. the peptide sequence is evaluated by an ALC (Average of Local Confidence) score. ALC is the average of the local confidence score of all the amino acids in the sequence.
b) Error (in ppm) is calculated by the software. comparing the exprimental m/z and the theoretical m/z (doubly-charged ion) derived from the amino acid deduced sequence.
c) The N-terminal methionine was in oxidized form.

d) The N-terminal glutamic acid residue was in the pyroglutamic acid form.

De novo interpretation of MS/MS Spectra

Peptide # 1

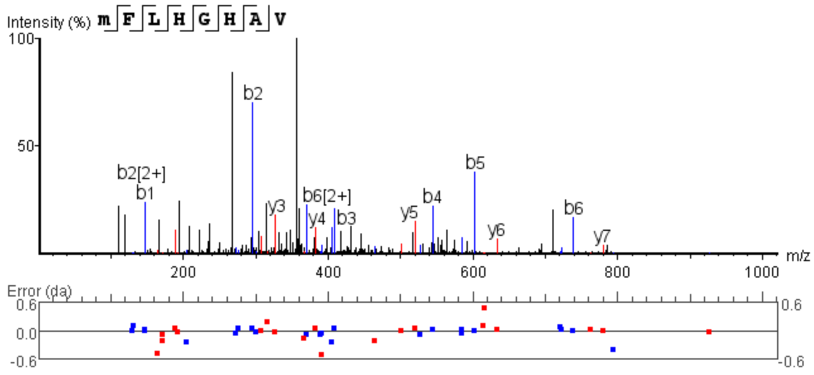

Ion Match | Survey

#	b	b-H2O	b-NH3	b (2+)	Seq	y	y-H2O	y-NH3	y (2+)	#
1	148.02	130.05	130.89	74.52	M(+15.99)					8
2	295.06	277.03	278.08	148.02	F	780.43	762.35	763.39	391.23	7
3	408.12	390.27	391.23	204.86	L	633.30	615.21	615.83	316.96	6
4	545.22	527.34	528.23	273.19	H	520.20	502.26	503.24	260.63	5
5	602.25	584.33	585.20	301.68	G	383.15	365.19	366.36	192.15	4
6	739.32	721.23	722.26	370.27	H	326.21	308.18	309.16	164.08	3
7	810.37	792.36	793.77	405.95	A	189.05	171.34	172.20	95.06	2
8					V	118.09	100.08	101.06	59.54	1

Peptide # 2

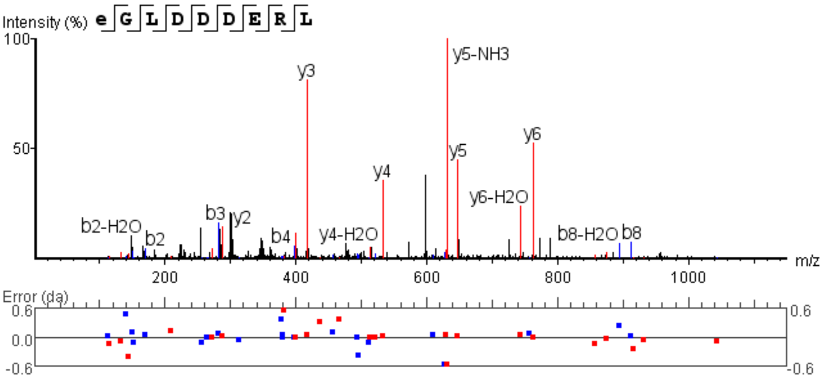

Ion Match | Survey

#	b	b-H2O	b-NH3	b (2+)	Seq	y	y-H2O	y-NH3	y (2+)	#
1	111.99	94.03	95.01	56.52	E(-18.01)					9
2	168.99	150.94	152.16	85.03	G	932.49	914.42	915.66	466.33	8
3	282.07	264.11	265.14	141.09	L	875.45	857.53	858.38	437.87	7
4	397.18	379.15	380.07	199.09	D	762.34	744.25	745.30	381.10	6
5	512.33	494.16	495.55	256.73	D	647.26	629.22	630.84	324.15	5
6	627.79	609.14	610.20	314.17	D	532.22	514.28	515.25	266.64	4
7	756.18	738.26	739.24	378.26	E	417.18	399.23	400.22	208.98	3
8	912.34	894.11	895.34	456.57	R	288.15	270.19	271.19	145.01	2
9					L	132.18	114.09	115.22	66.55	1

Peptide # 3

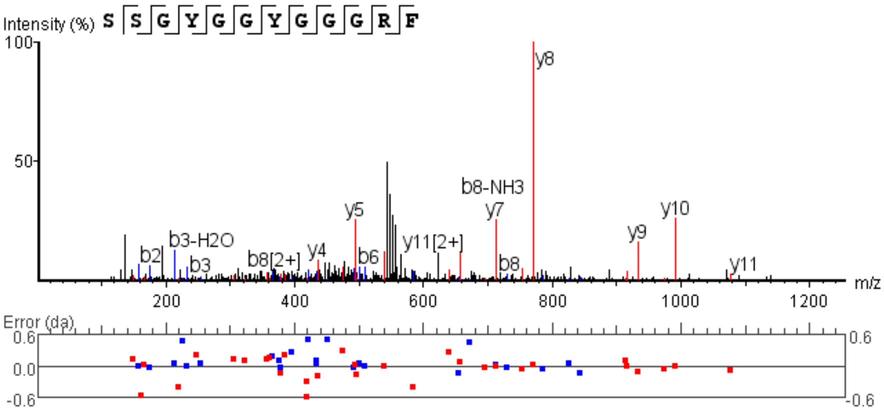

Ion Match | Survey

#	b	b-H2O	b-NH3	b (2+)	Seq	y	y-H2O	y-NH3	y (2+)	#
1	88.04	70.03	71.01	44.52	S					12
2	175.12	157.05	158.04	88.04	S	1077.56	1059.46	1060.45	539.22	11
3	232.08	214.01	215.07	116.55	G	990.44	972.43	973.48	495.91	10
4	394.88	377.01	378.16	198.08	Y	933.53	915.29	916.38	467.21	9
5	451.67	434.04	435.08	226.09	G	770.33	752.35	753.40	385.46	8
6	509.20	491.22	492.17	255.05	G	713.31	695.33	696.35	357.03	7
7	671.80	654.25	655.37	336.63	Y	656.23	638.30	639.00	328.66	6
8	729.33	711.27	712.22	364.95	G	493.22	474.92	476.22	246.90	5
9	786.36	768.30	769.28	393.65	G	436.43	418.54	419.80	219.03	4
10	843.49	825.32	826.24	421.65	G	379.36	361.20	362.00	190.10	3
11	999.43	981.42	982.40	500.14	R	322.08	304.18	305.02	162.16	2
12					F	166.06	148.08	148.91	83.54	1

Peptide # 4

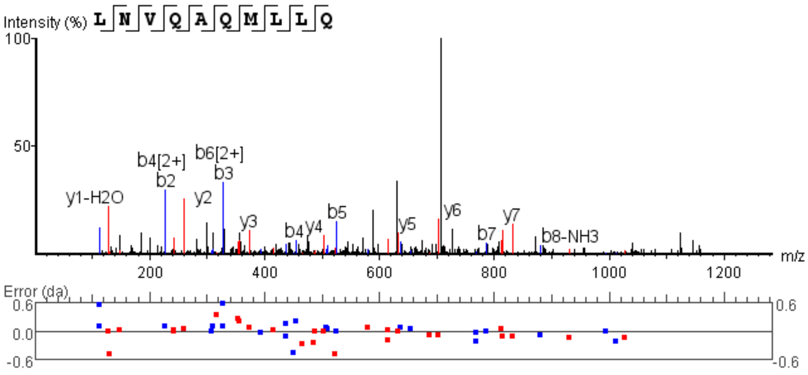

#	b	b-H2O	b-NH3	b (2+)	Seq	y	y-H2O	y-NH3	y (2+)	#
1	113.99	96.08	97.06	57.55	L					10
2	228.01	210.12	211.11	113.99	N	1044.55	1026.54	1027.68	523.27	9
3	327.08	309.18	310.04	164.10	V	930.65	912.50	913.48	466.04	8
4	455.03	437.37	438.06	228.01	Q	831.55	813.35	814.54	416.19	7
5	526.28	508.20	509.19	263.65	A	703.46	685.37	686.45	352.19	6
6	654.30	636.25	637.33	327.08	Q	632.32	614.53	615.27	316.32	5
7	785.40	767.61	768.42	393.23	M	504.28	486.54	487.24	252.64	4
8	898.48	880.47	881.54	450.21	L	373.14	354.96	355.99	187.12	3
9	1011.81	993.56	994.53	506.28	L	260.10	242.12	243.14	131.07	2
10					Q	147.03	129.07	130.05	74.04	1

Peptide # 5

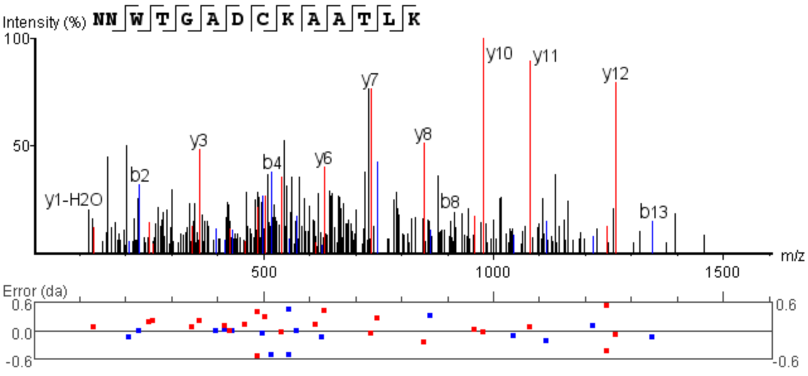

#	b	b-H2O	b-NH3	b (2+)	Seq	y	y-H2O	y-NH3	y (2+)	#
1	115.05	97.04	98.02	58.03	N					14
2	229.11	211.08	212.07	115.05	N	1378.68	1360.67	1361.65	689.84	13
3	415.14	397.16	398.15	208.23	W	1264.74	1247.06	1247.06	632.82	12
4	516.74	498.29	499.25	258.61	T	1078.46	1060.55	1061.53	539.81	11
5	573.23	555.75	555.75	287.12	G	977.55	959.47	960.48	489.25	10
6	644.28	626.41	627.25	322.64	A	920.49	902.48	903.46	460.59	9
7	759.31	741.30	742.28	380.15	D	849.70	831.44	832.42	425.23	8
8	861.97	844.30	845.29	431.67	C	734.49	716.41	717.40	367.71	7
9	990.41	972.40	973.38	495.71	K	630.99	613.40	614.24	316.21	6
10	1061.45	1043.57	1044.42	531.22	A	503.00	485.87	485.87	251.97	5
11	1132.48	1114.47	1115.69	566.74	A	432.28	414.27	415.14	216.64	4
12	1233.53	1215.39	1216.51	617.27	T	361.03	343.23	344.12	181.12	3
13	1346.76	1328.61	1329.59	673.81	L	259.97	242.19	243.17	130.60	2
14					K	147.11	129.02	130.09	74.06	1

Peptide # 6

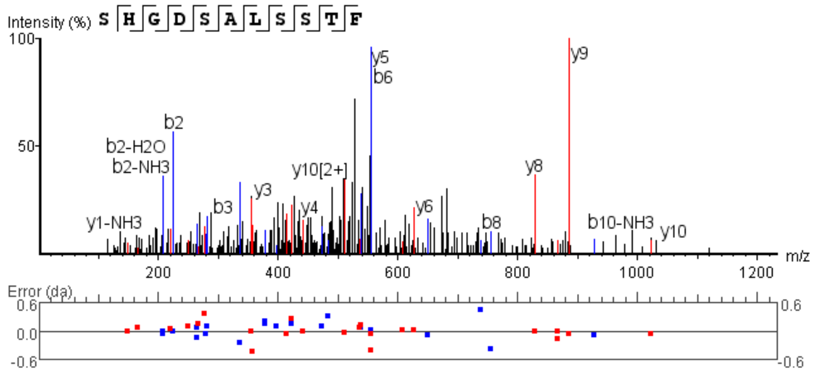

Ion Match	Survey									
#	b	b-H2O	b-NH3	b (2+)	Seq	y	y-H2O	y-NH3	y (2+)	#
1	88.04	70.03	71.01	44.52	S					11
2	225.10	207.16	208.09	113.05	H	1021.54	1003.45	1004.43	511.27	10
3	282.01	264.25	264.99	141.56	G	884.48	866.55	867.36	442.70	9
4	397.04	378.90	380.12	199.07	D	827.38	809.37	810.35	414.26	8
5	483.86	466.17	467.15	242.59	S	712.35	694.34	695.32	357.12	7
6	555.18	537.11	538.19	278.17	A	625.28	607.27	608.29	313.16	6
7	668.30	650.38	651.27	334.90	L	554.34	536.17	537.11	277.26	5
8	755.72	736.87	738.31	377.98	S	441.19	422.92	424.17	221.04	4
9	842.36	824.35	825.34	421.51	S	354.18	336.16	337.14	177.58	3
10	943.41	925.40	926.47	472.07	T	266.96	249.12	249.99	134.07	2
11					F	166.00	148.08	149.04	83.54	1

Peptide # 7

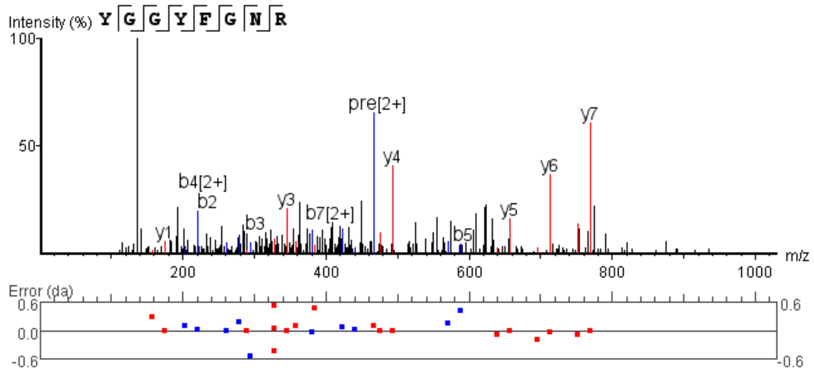

Ion Match	Survey									
#	b	b-H2O	b-NH3	b (2+)	Seq	y	y-H2O	y-NH3	y (2+)	#
1	164.07	146.06	147.04	82.54	Y					8
2	221.04	203.08	203.93	111.05	G	770.37	752.43	753.33	385.18	7
3	277.92	260.10	261.08	139.56	G	713.39	695.33	696.50	357.06	6
4	441.14	423.06	424.15	221.04	Y	656.33	638.30	639.38	328.60	5
5	587.82	570.05	571.22	295.18	F	493.27	475.24	476.23	247.13	4
6	645.27	627.26	628.24	323.13	G	346.20	328.60	328.60	173.59	3
7	759.31	741.30	742.28	380.19	N	289.17	271.15	272.13	145.08	2
8					R	175.10	156.81	158.09	88.06	1

Peptide # 8

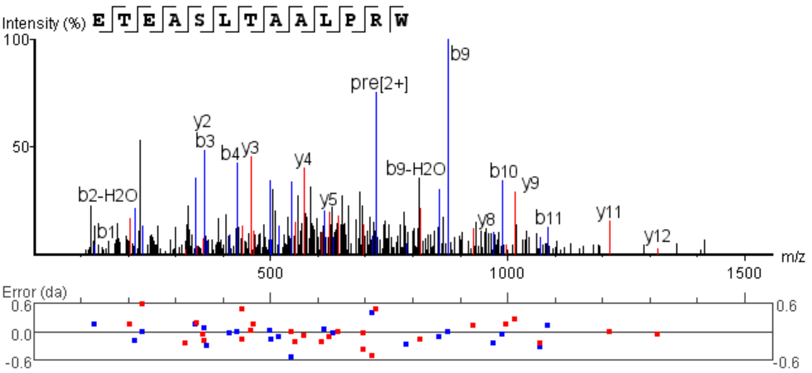

Ion Match | Survey

#	b	b-H2O	b-NH3	b (2+)	Seq	y	y-H2O	y-NH3	y (2+)	#
1	129.89	112.04	113.02	65.53	E					13
2	231.10	213.30	214.07	116.05	T	1315.77	1297.69	1298.67	658.35	12
3	360.04	341.96	343.11	180.57	E	1214.67	1196.64	1197.63	608.06	11
4	431.17	413.21	414.15	216.09	A	1085.61	1067.86	1068.58	543.32	10
5	518.32	500.15	501.37	259.60	S	1014.29	996.40	997.55	507.79	9
6	631.34	613.23	614.21	316.15	L	927.38	909.53	910.51	464.09	8
7	732.34	713.92	715.31	366.98	T	814.63	796.45	797.43	407.73	7
8	803.38	785.64	786.35	402.19	A	713.92	695.44	696.78	357.26	6
9	874.40	856.53	857.39	437.71	A	642.36	624.48	625.35	321.93	5
10	987.55	969.49	970.72	494.25	L	571.43	553.56	554.31	286.17	4
11	1084.42	1066.54	1067.86	543.32	P	458.20	439.74	441.40	229.04	3
12	1240.65	1222.64	1223.63	620.83	R	361.39	343.19	343.98	181.10	2
13					W	204.93	187.09	188.07	103.05	1

Peptide # 9

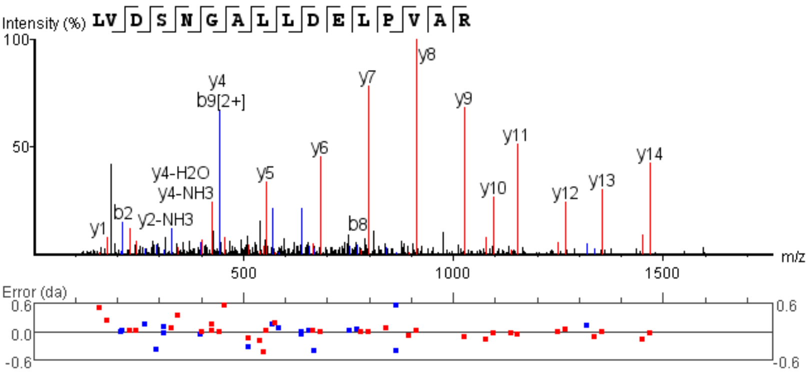

Ion Match | Survey

#	b	b-H2O	b-NH3	b (2+)	Seq	y	y-H2O	y-NH3	y (2+)	#
1	114.09	96.08	97.06	57.55	L					16
2	213.12	195.15	196.13	107.08	V	1568.83	1550.82	1551.80	784.91	15
3	328.10	310.22	311.04	164.59	D	1469.81	1451.92	1452.73	735.38	14
4	415.22	397.29	398.19	208.13	S	1354.75	1336.72	1337.82	677.87	13
5	529.26	511.59	512.24	264.97	N	1267.63	1249.69	1250.68	634.35	12
6	586.20	568.27	569.09	294.04	G	1153.71	1135.65	1136.66	577.14	11
7	657.29	639.38	640.31	329.16	A	1096.68	1078.80	1079.61	549.27	10
8	770.33	752.39	753.33	385.70	L	1025.72	1007.59	1008.57	513.45	9
9	883.49	865.88	865.88	442.25	L	912.49	894.50	895.59	456.20	8
10	998.52	980.51	981.49	499.76	D	799.42	781.43	782.40	400.21	7
11	1127.56	1109.55	1110.53	564.28	E	684.42	666.34	667.38	342.70	6
12	1240.64	1222.63	1223.62	620.82	L	555.32	537.35	538.54	278.18	5
13	1337.82	1319.53	1320.67	669.77	P	442.25	424.10	425.20	221.64	4
14	1436.76	1418.75	1419.74	718.88	V	344.86	327.21	328.10	173.11	3
15	1507.80	1489.79	1490.77	754.40	A	246.11	228.15	229.10	123.58	2
16					R	174.88	156.58	158.09	88.06	1

Peptide # 10

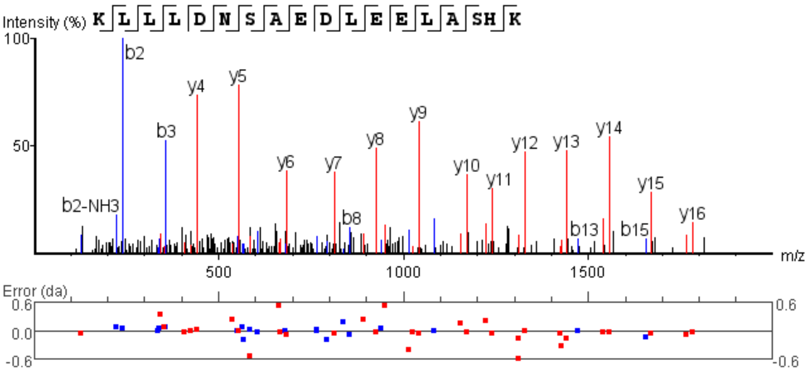

#	b	b-H2O	b-NH3	b (2+)	Seq	y	y-H2O	y-NH3	y (2+)	#
1	129.17	111.09	112.08	65.05	K					18
2	242.12	224.18	225.07	121.59	L	1896.95	1878.94	1879.93	948.42	17
3	355.17	337.27	338.18	178.14	L	1783.91	1765.86	1766.94	892.19	16
4	468.35	450.34	451.33	234.68	L	1670.84	1652.78	1653.76	835.89	15
5	583.35	565.28	566.55	292.19	D	1557.75	1539.69	1540.72	779.35	14
6	697.42	679.40	680.40	349.21	N	1442.86	1424.73	1425.97	721.84	13
7	784.46	766.42	767.39	392.73	S	1328.63	1310.78	1312.20	664.26	12
8	855.58	837.29	838.47	428.25	A	1241.66	1223.59	1224.34	621.30	11
9	984.54	966.53	967.51	492.77	E	1170.60	1152.55	1153.37	586.33	10
10	1099.56	1081.55	1082.52	550.27	D	1041.59	1023.54	1024.49	521.26	9
11	1212.65	1194.64	1195.62	606.88	L	926.53	908.48	909.47	463.75	8
12	1341.69	1323.68	1324.66	671.35	E	813.47	795.40	796.38	407.23	7
13	1470.72	1452.72	1453.71	735.87	E	684.47	666.38	667.34	342.33	6
14	1583.82	1565.81	1566.79	792.61	L	555.33	537.31	538.03	278.16	5
15	1654.99	1636.84	1637.83	827.93	A	442.20	424.23	425.21	221.62	4
16	1741.89	1723.88	1724.86	871.44	S	371.20	353.19	354.07	186.10	3
17	1878.94	1860.93	1861.92	939.91	H	284.17	266.16	267.14	142.59	2
18					K	147.11	129.17	130.09	74.06	1

Peptide # 11

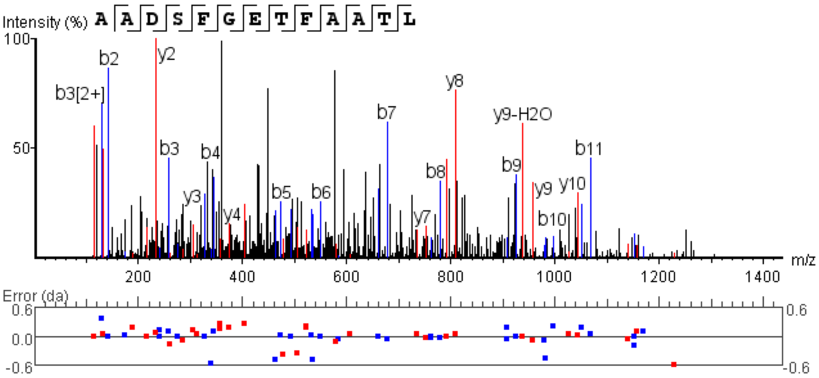

Ion Match Survey

#	b	b-H2O	b-NH3	b (2+)	Seq	y	y-H2O	y-NH3	y (2+)	#
1	72.04	54.03	55.02	36.52	A					13
2	143.06	125.07	126.06	72.04	A	1230.17	1211.56	1212.54	615.28	12
3	257.98	240.08	240.94	129.16	D	1158.40	1140.59	1141.50	579.88	11
4	345.02	327.12	328.13	173.03	S	1043.47	1025.49	1026.41	522.06	10
5	492.19	474.15	475.18	246.60	F	956.55	938.47	939.45	479.13	9
6	549.22	531.19	532.20	275.11	G	809.33	791.40	792.38	404.92	8
7	678.35	660.28	661.25	340.21	E	752.43	734.31	735.36	376.69	7
8	779.37	761.29	762.34	390.16	T	623.34	605.33	606.25	312.09	6
9	926.40	908.46	909.16	464.19	F	522.06	504.64	505.27	261.83	5
10	997.20	979.52	980.86	499.21	A	375.02	356.94	358.04	187.93	4
11	1068.40	1050.26	1051.44	535.21	A	304.04	286.27	287.16	152.59	3
12	1169.40	1151.48	1152.68	585.31	T	233.06	215.12	216.12	117.07	2
13					L	132.02	114.09	115.06	66.55	1

Peptide # 12

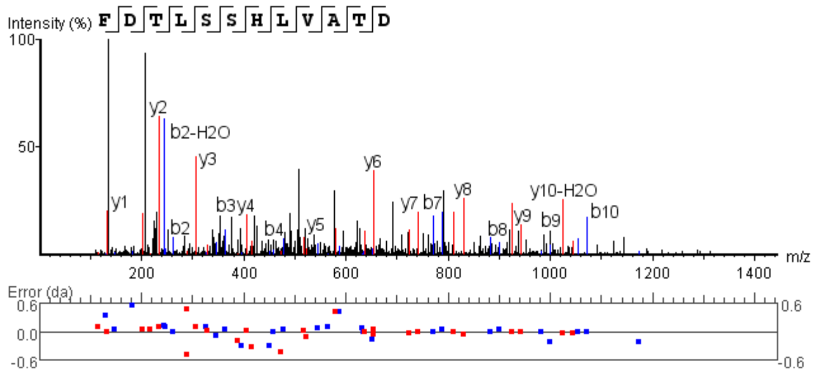

Ion Match | Survey

#	b	b-H2O	b-NH3	b (2+)	Seq	y	y-H2O	y-NH3	y (2+)	#
1	148.02	130.07	130.68	74.54	F					12
2	263.11	244.96	245.95	132.05	D	1158.56	1140.55	1141.54	579.33	11
3	364.08	346.23	347.12	181.99	T	1043.58	1025.57	1026.51	522.38	10
4	477.18	459.20	460.21	239.12	L	942.46	924.49	925.46	472.19	9
5	564.16	546.16	547.24	282.63	S	829.48	811.39	812.38	415.52	8
6	651.47	633.18	634.26	326.04	S	742.38	724.40	725.35	371.69	7
7	788.30	770.36	771.33	394.99	H	655.28	637.35	638.31	328.13	6
8	901.38	883.43	884.41	451.52	L	518.24	500.27	501.26	259.64	5
9	1000.74	982.49	983.48	500.76	V	405.15	387.19	388.36	203.04	4
10	1071.56	1053.52	1054.52	536.27	A	306.01	288.60	288.60	153.56	3
11	1172.83	1154.58	1155.57	586.35	T	234.98	217.01	218.07	118.05	2
12					D	134.03	115.92	117.02	67.52	1

Peptide # 13

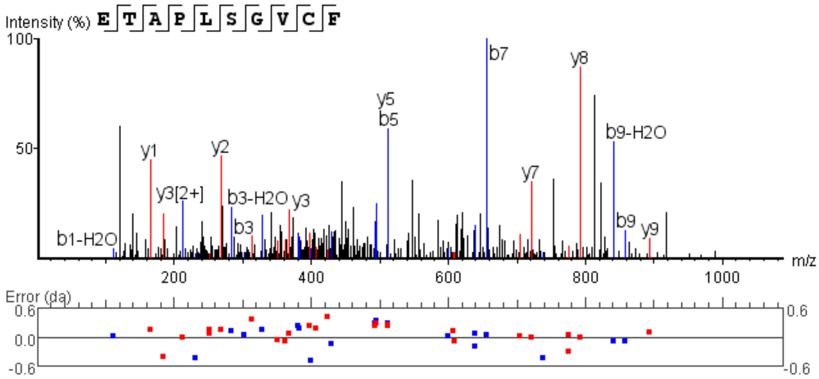

Ion Match | Survey

#	b	b-H2O	b-NH3	b (2+)	Seq	y	y-H2O	y-NH3	y (2+)	#
1	130.05	111.99	113.02	65.53	E					10
2	231.54	213.09	214.07	116.05	T	894.32	876.43	877.41	447.72	9
3	302.07	283.98	285.11	151.57	A	793.40	775.30	776.68	396.93	8
4	399.69	380.93	381.95	200.09	P	722.35	704.30	705.33	361.76	7
5	511.96	493.95	494.90	256.64	L	625.30	607.14	608.36	312.75	6
6	599.27	581.29	582.28	300.15	S	511.96	493.95	494.90	256.61	5
7	656.26	638.53	639.19	328.50	G	424.74	407.17	407.96	213.09	4
8	755.39	737.38	738.81	378.20	V	368.07	350.21	351.14	185.01	3
9	858.50	840.48	841.38	429.84	C	268.93	250.91	251.98	135.05	2
10					F	165.90	148.08	149.06	83.54	1

Peptide # 14

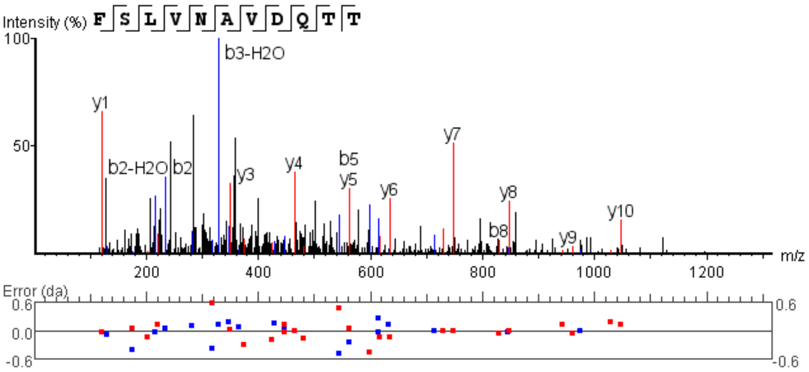

Ion Match | Survey

#	b	b-H2O	b-NH3	b (2+)	Seq	y	y-H2O	y-NH3	y (2+)	#
1	148.08	130.15	131.05	74.54	F					11
2	235.05	217.13	218.08	118.05	S	1047.38	1029.32	1030.50	524.27	10
3	348.00	330.03	331.17	175.01	L	960.58	942.49	943.33	480.93	9
4	447.22	429.09	430.23	224.13	V	847.40	829.41	830.46	424.40	8
5	561.57	543.29	544.77	281.05	N	748.36	730.36	731.32	374.98	7
6	632.18	614.05	615.35	317.06	A	634.46	616.29	617.43	317.06	6
7	731.41	713.40	714.36	366.11	V	563.20	544.77	546.24	282.13	5
8	846.47	828.43	829.41	423.72	D	464.18	446.03	447.22	232.60	4
9	974.47	956.48	957.47	487.75	Q	349.14	331.16	332.14	175.01	3
10	1075.54	1057.53	1058.52	538.27	T	220.97	203.26	204.09	111.06	2
11					T	120.11	102.05	103.04	60.53	1
